# Endocrine risk factors for COVID-19: Endogenous and exogenous glucocorticoid excess

**DOI:** 10.1007/s11154-021-09670-0

**Published:** 2021-07-09

**Authors:** Frederick Vogel, Martin Reincke

**Affiliations:** Medizinische Klinik und Poliklinik IV, Klinikum der Universität, Ludwig-Maximilians-Universität München, Munich, Germany

**Keywords:** COVID-19, Hypercortisolism, Glucocorticoids, Cortisol, Cushing’s syndrome

## Abstract

Patients with endogenous or exogenous glucocorticoid (GC) excess exhibit a range of side effects, including an increased risk of infections. Via both mechanism, immune impairments and cardiometabolic concomitant diseases, patients with GC excess could be at increased risk for COVID-19. The impact on incidence and outcome of a SARS-CoV-2 infection in this population are not yet completely clear. This review aims to compile the data available to date and to discuss the existing literature on this topic. Further we highlight potential effects of SARS-CoV-2 on the hypothalamic-pituitary-adrenal axis as well as the influence of endogenous or exogenous GC excess on SARS-CoV-2 mRNA vaccination. There is growing evidence suggesting an increased risk of infection and severe outcome in patients with high-dose GC therapy after contracting SARS-CoV-2. The few data and case reports on patients with endogenous GC excess and SARS-CoV-2 infection point in a similar direction: chronic GC excess seems to be associated with an unfavorable course of COVID-19. Whether this is mainly a primary immune-mediated effect, or also triggered by the many GC-associated comorbidities in this population, is not yet fully understood. Patients with endogenous or exogenous GC excess should be considered as a vulnerable group during the SARS-CoV-2 pandemic. Regardless of the cause, vaccination and consistent surveillance and control of associated comorbidities are recommended.

## Introduction

The novel coronavirus, named severe acute respiratory syndrome coronavirus 2 (SARS-CoV-2), was first identified in January 2020. Since the initial reports of a cluster of pneumonia cases in Wuhan, China, in December 2019, acute infection by SARS-CoV-2, officially named as coronavirus disease-19 (COVID-19), has spread throughout the world [1]. Symptoms and clinical course of SARS-CoV-2 infection are highly variable, ranging from asymptomatic to lethal [2, 3]. The symptomatology and impairments of COVID-19 primarily affect the respiratory tract, but many other organs are also involved, such as the gastrointestinal tract, liver, kidneys, skin, endocrine organs as well as the musculoskeletal, central nervous and cardiovascular system [4–6]. Epidemiological evidence from the first cases of hospitalized COVID-19 patients demonstrated that COVID-19 more likely affects older males with chronic underlying conditions, mainly metabolic, cardiovascular and cerebrovascular diseases [7]. In further studies, older age and a large body of comorbidities have been found to be associated with morbidity and mortality due to COVID-19 [2, 8]. But the underlying mechanisms remain largely unknown [9]. Especially patients with chronic metabolic diseases, like obesity, diabetes, hypertension, cardiovascular and kidney diseases showed a higher risk of acute respiratory distress syndrome (ARDS) and fatal outcome [10]. Administration of dexamethasone has been shown to be effective therapy in patients with respiratory complications due to COVID-19 [11, 12]. This appears to be partly contradictory, as the use of exogenous glucocorticoids (GCs) is, despite their efficiency in terms of immunomodulation, frequently associated with concomitant metabolic diseases, such as obesity, hypertension, cardiovascular diseases, hepatic steatosis, insulin resistance and type 2 diabetes [13–15]. In patients with endogenous GC excess, a serious endocrine disease caused by chronic, autonomous, and excessive secretion of cortisol from the adrenal glands, the metabolic side-effects of GCs are often many times more pronounced. While chronic endogenous hypercortisolism, i.e. endogenous Cushing’s syndrome (CS), is a rare disorder with an annual incidence of 2-3/million [16], up to 70% of patients under GC treatment experience adverse systemic side-effects in the sense of an iatrogenic CS [15, 17]. Regarding the latter, it is estimated that 1-3% of the population of the United States, United Kingdom or Denmark take GCs for the treatment of inflammatory and autoimmune diseases [18–20]. Beside the metabolic changes, patients with both endogenous and iatrogenic hypercortisolism also have increased risk of various bacterial, viral, fungal and parasitic infections, induced by an immunocompromised state [21–23]. The outcomes of SARS-CoV-2 infection in patients with immune-modulatory therapies are, so far, only partly understood. Understanding the real impact of GC treatment on the frequency and severity of COVID-19 is an important inter-disciplinary issue. In this article, we will review the epidemiology and outcomes of SARS-CoV-2 infections in patients with endogenous and exogenous GC excess based on the literature available to date. Furthermore, we discuss safety and efficacy of SARS-CoV-2 mRNA vaccines in patients with GC excess as well as the potential effects of a SARS-CoV-2 infection on the hypothalamic-pituitary-adrenal (HPA) axis.

## Risk in exogenous glucocorticoid excess

Since their discovery in the 1940s and the recognition of the immunosuppressive actions, GCs have been considered one of the most widely used and effective therapies to control inflammatory and autoimmune diseases [24]. Patients receiving chronic oral GCs due to rheumatic diseases are at higher risk for serious infections [25]. Over the past 12 months, since the outbreak of SARS-CoV-2, an unprecedented number of case reports and studies on COVID-19 has been published, and numerous risk factors for severe disease have been identified. Whether or not patients with systemic GC treatment are at an increased risk of SARS-CoV-2 infection and/or severe outcomes is so far not fully clarified. On the one hand, GCs were discussed theoretically to mitigate hyper-immune reactions responsible for severe disease progression [26]. An enhanced local innate immune defense in respiratory epithelial cells was reported in response to GC exposure, beside the known anti-inflammatory and anti-allergic actions of GCs [27]. However, chronic GC exposure might also increase the risk for infection and/or severe COVID-19 in these patients. Because of the mostly underlying immune system dysfunction, the treatment with immunosuppressive drugs and a higher level of comorbidities, these patients belong to a vulnerable population. Indeed, a meta-analysis of patients with COVID-19 and autoimmune diseases demonstrated in a meta-regression analysis a higher prevalence of COVID-19 in studies with a higher proportion of GC use in patients with autoimmune diseases (regression coefficient: 0.020, 95% CI 0.001 to 0.040, p = 0.042) [28]. Due to the heterogeneous studies with different sample sizes, variable diseases included, diagnostic criteria and geographic location, these data should be interpreted with caution. However, there are other studies of patients with autoimmune or inflammatory diseases and GC exposure that have described an increased risk of SARS-CoV-2 infection [26, 29, 30]. Table 1 shows studies with patients under GC treatment and SARS-CoV-2 infection. Concerning the prevalence of SARS-CoV-2 infection, in all three studies mentioned, higher GC doses were associated with a higher risk of contracting SARS-CoV-2. As an exception, Soldevila-Demenech and colleagues reported that women treated with prednisone-equivalent doses ≤ 10 mg per day had a decreased adjusted relative risk for symptoms of SARS-CoV-2. However, since the primary outcome of the study was the clinical diagnosis of COVID-19, and the majority of patients did not undergo a confirmatory SARS-CoV-2 test, these data should be interpreted cautiously. Overall, they also found an increased risk when prednisone-equivalent doses were > 10 mg per day [30].

**Table 1:** Patients with pre-existing exogenous GC treatment and SARS-CoV-2 infection

First author	Year of publication	Patient cohort	Number of patients	Number of hospitalized patients	Risk for infection / hospitalization / severe outcome	Key messages/ authors’ conclusion
Glucocorticoids and the risk of infection						
Favalli et al. [26]	2020	Patients with chronic inflammatory arthritis	641 with GC treatment, of which 13 had a SARS-CoV-2 infection	unknown	• Increased risk for infection, OR 2.89 (95%CI 1.26 to 6.62, p=0.01); prednisone dose <2.5mg/day OR 1.41 (95%CI 0.39 to 5.15, p=0.61); prednisone dose ≥2.5mg/day OR 4.22 (95%CI 1.74 to 10.23, p=0.001)	• Use of GCs (prednisone doses ≥2.5mg/day) increased the risk of SARS-CoV-2 infection • Use of GCs independently predict increased risk of SARS-CoV-2 infection • Use of GCs should be cautiously evaluated during the pandemic
Fitzgerald et al. [29]	2021	Patients with autoimmune or inflammatory conditions	1120 with GC treatment, 85 of whom with SARS-CoV-2 infection	unknown	• GC exposure in the past year was associated with 43% increased odds of COVID-19 in multivariable-adjusted models (OR 1.43, 95%CI 1.08 to 1.89) • Higher odds for COVID-19 in individuals with high prednisone equivalent doses: 7.5 to 60mg/day OR 1.60 (95%CI 1.05 to 2.44); >60mg/day OR 1.95 (95%CI 0.87 to 4.39)	• Exposure to GCs may increase risk of contracting COVID-19 • A potential non-linear association between total GC dose and COVID-19 risk was noted
Soldevila-Demenech et al. [30]	2020	Patients with immunomediated inflammatory diseases	527 with GC treatment, 35 with symptoms of SARS-CoV-2 infection	unknown	• Adjusted relative risk for COVID-19 symptoms in patients with GC treatment ≤10mg/day 0.94 (95%CI 0.61 to 1.43) and >10mg/day 1.76 (95%CI 0.90 to 3.45) • Adjusted relative risk for COVID-19 symptoms in women with low GC treatment ≤10mg/day 0.72 (95%CI 0.42 to 1.22)	• Treatment with low GC doses decreased the incidence of COVID-19 predominantly in women, whereas high doses seemed to produce the opposite effect
Glucocorticoids and the risk of hospitalization						
Gianfrancesco et al. [31]	2020	Patients with rheumatic diseases	189 with GC treatment and SARS-CoV-2 infection	110	• Higher proportion of patients receiving high doses of glucocorticoids in hospitalized patients vs non-hospitalized patients (16% vs 7% for doses ≥10mg/day, p=0.01) • GC treatment (prednisone-equivalent doses ≥10mg/day) was associated with higher odds of hospitalization (adjusted OR 2.05, 95%CI 1.06 to 3.96, p=0.03); odds for patients with prednisolone-equivalent doses 1-9mg/day: OR 1.03, 95%CI 0.64 to 1.66, p=0.91	• GC use at a prednisone-equivalent dose ≥10mg/day was associated with a higher odds of hospitalization
Haberman et al. [32]	2020	Patients with immune-mediated inflammatory diseases	8 with GC treatment and SARS-CoV-2 infection; overall 86 patients with SARS-CoV-2 infection	4	• GC use was higher among patients hospitalized (4 of 14, 29%) than those that were not hospitalized (4 of 72, 6%)	• The use of oral GCs was higher among patients with immune-mediated inflammatory disease for whom hospitalization was warranted
Haberman et al. [33]	2020	Patients with inflammatory arthritis	13 with GC treatment and SARS-CoV-2 infection; overall 103 patients with SARS-CoV-2 infection	10	• Chronic GC treatment was more common in patients requiring hospitalization (37% hospitalized vs 4% ambulatory, p<0.001) • Risk of hospitalization in patients with oral GCs: adjusted OR 21.2 (95%CI 4.09 to 109.03, p<0.001)	• Patients with inflammatory arthritis and GC treatment are at higher risk for hospitalization after contracting SARS-CoV-2 • COVID-19 outcomes were worse in patients receiving oral GCs
Arleo et al. [34]	2021	Patients with rheumatic diseases	37 with oral GC treatment and SARS-CoV-2 infection; overall 70 patients with SARS-CoV-2 infection	24	• Hospitalized patients for COVID-19 used oral GC more frequently than those treated as outpatients (71% vs 36%, p<0.01) • Out of 24 patients with oral GC treatment that were hospitalized for COVID-19, 6 patients died (5 patients with >10mg/day) • All patients who died in the study cohort (6/70) used oral GCs	• Patients with rheumatic diseases and chronic GCs were hospitalized more frequently • Chronic GC use increased COVID-19 disease severity
Hasseli et al. [35]	2021	Patients with inflammatory rheumatic and musculoskeletal diseases	180 with GC treatment and SARS-CoV-2 infection	77	• Higher odds for hospitalization in GC treated patients (dosages >5mg/day) by multivariable logistic regression: OR 3.67, 95%CI 1.49 to 9.05, p=0.005; dosages ≤5mg/day OR 1.38, 95%CI 0.80 to 2.37, p=0.25 • Of 77 hospitalized patients, 18 needed invasive ventilation	• Current or prior treatment with GCs was a major and independent risk factor for hospitalization • Since uncontrolled rheumatic and musculoskeletal disease activity also enhances the risk of infections, the results should not encourage stopping GC therapy • GCs should be administered in the lowest possible dose
Fredi et al. [36]	2020	Patients with rheumatic and musculoskeletal diseases	43 with GC treatment and SARS-CoV-2 infection; overall 65 patients with SARS-CoV-2 infection	31	• Cases that required admission to hospital vs those that were not admitted to hospital showed no significant difference in background therapy and comorbidities • No differences in therapies between deceased patients and survivors	• A poor outcome from COVID-19 seems to be associated with older age and the presence of comorbidities rather than the type of rheumatic disease or background therapy
Freites Nunez et al. [37]	2020	Patients with autoimmune inflammatory rheumatic diseases	61 with GC treatment and SARS-CoV-2 infection; overall 123 patients with SARS-CoV-2 infection	29	• OR of hospital admission related to COVID-19 in patients with GC treatment by univariate analysis 2.01 (95%CI 0.97 to 4.13, p=0.05) • GC exposure showed no significant difference (probability of hospital admission) in the multivariable analysis (adjusted OR 1.97, 95%CI 0.77 to 5.01, p=0.15)	• No statistically significant findings for exposure to disease-modifying antirheumatic drugs were found in the final multiple regression model
Cordtz et al. [38]	2020	Patients with rheumatoid arthritis	2411 with GC therapy, of which 5 were hospitalized with COVID-19; unknown how many with (ambulatory) SARS-CoV-2 infection	5	• Absolute standardized risk for hospitalization at 60 days 0.16 (95%CI 0.03 to 0.33) • Adjusted HR for hospitalization 1.22 (95%CI 0.47 to 3.15)	• Patients with inflammatory rheumatic diseases had higher incidence of COVID-19 hospitalization • Treatment with GCs was not associated with hospitalization
Glucocorticoids and the risk of severe course of disease/ COVID-19-related death						
Brenner et al. [40]	2020	Patients with inflammatory bowel disease	37 with systemic GC treatment and SARS-CoV-2 infection	26	• Out of 37 patients with oral GC treatment, 9 patients (24%) had a severe course of disease (ICU/ventilator/death) • Adjusted OR for ICU/vent/death 6.87 (95%CI 2.30 to 20.51, p<0.001) • Adjusted OR for COVID-19-related death 11.62 (95%CI 2.09 to 64.74, p=0.005)	• GC use was a strong risk factor for adverse COVID-19 outcomes and positively associated with the outcome of death • Steroid-sparing treatments will be important managing patients with inflammatory bowel disease through this pandemic
Strangfeld et al. [41]	2021	Patients with rheumatic diseases	1273 with GC treatment and SARS-CoV-2 infection; overall 3729 patients with SARS-CoV-2 infection	unknown	• 217 of 1273 patients with GC treatment deceased • Adjusted OR for COVID-19-related death in patients with prednisolone-equivalent dose >10mg/day: OR 1.69 (95%CI 1.18 to 2.41) • Adjusted OR for COVID-19-related death in patients with prednisolone-equivalent dose ≤10mg/day: OR 1.43 (95%CI 0.98 to 2.09)	• Treatment with higher dosages of glucocorticoids (>10mg/day prednisolone-equivalent dose) was an independent factor associated with COVID-19-related death • Patients with higher disease activity have higher odds of COVID-19-related death
Rutherford et al. [42]	2021	Patients with systemic vasculitis	45 with GC treatment and SARS-CoV-2 infection	45	• 22 patients (49%) with GC therapy experienced a severe outcome (advanced oxygen therapy/ventilation/death) • Adjusted OR for severe outcome in patients with GC therapy: OR 3.7 (95%CI 1.1 to 14.9, p=0.047)	• GC therapy at presentation was associated with severe outcome in COVID-19 • GC exposure remained a poor prognostic indicator even after adjusting for vasculitis disease activity • Patients who are receiving GCs should be closely monitored when presenting with COVID-19 since their risk of progression to a severe state appears higher
Pablos et al. [43]	2020	Hospitalized patients with rheumatic diseases	91 with GC treatment and SARS-CoV-2 infection; overall 228 patients with SARS-CoV-2 infection	91	• GC therapy in hospitalized patients was associated with poorer outcome by bivariable analysis (OR 2.20, 95%CI 1.36 to 3.54, p=0.001) • No association of antirheumatic therapies and a severe COVID-19 outcome in the multivariable adjusted analysis (GC treatment: OR 1.10, 95%CI 0.60 to 2.01, p=0.76)	• Immunosuppressive therapies do not significantly greater the risk for poor outcomes
Ye et al. [44]	2021	Patients with connective tissue diseases	22 with GC treatment and SARS-CoV-2 infection; overall 48 with SARS-CoV-2 infection	22	• Patients on low- to medium-dose GCs (5–15mg/day prednisone) had less severe/critical conditions compared to those without GC use before diagnosis (6/22 vs 15/26, p<0.05)	• Low- to medium-dose GCs may reduce the progression of COVID-19 from mild/moderate conditions to severe/critical conditions

*GC* glucocorticoid, *SARS-CoV-2* severe acute respiratory syndrome coronavirus 2, *COVID-19* coronavirus disease-19, *OR* odds ratio, *HR* hazard ratio, *CI* confidence interval, *vs* versus

The risk for hospitalization due to COVID-19 was analyzed by Gianfrancesco and colleagues in a publication from the COVID-19 Global Rheumatology Alliance that analyzed factors associated with hospitalization in 600 cases of COVID-19 in patients with rheumatic diseases [31]. They found, that the use of high-dose GCs (≥ 10mg per day of prednisone-equivalent) was associated with hospitalization (adjusted OR 2.05, 95% CI 1.06 to 3.96, p = 0.03). Compared to non-hospitalized patients, hospitalized patients due to COVID-19 were more likely to have chronic GC therapy (16% vs 7% for doses ≥ 10mg per day, p = 0.01). In multivariate regression models, age, comorbidities as well as GC therapy prior to the SARS-CoV-2 infection remained associated with the risk of hospitalization [31]. Other studies and case reports of patients with immune-mediated inflammatory diseases are in line with these results and point to similar observations (Table 1): the use of GCs is higher among hospitalized patients, and chronic oral GC use is associated with a higher risk of hospitalization due to COVID-19 in this population [32–35]. In contrast, other studies and case reports of patients with rheumatic diseases showed no direct and significant relation between GC use and the risk of a severe course of disease [36–38]. In general, the outcome for patients with rheumatic diseases does not seem to differ from that of the general population when demographic factors and comorbidities are taken into account [39]. However, since medication use and outcomes are most likely associated [31], GC administration *per se* already has many unfavorable side effects, and patients with underlying rheumatic diseases are more likely to have comorbidities, this should be considered and the data interpreted carefully.

The risk for a severe course of disease or death due to COVID-19 was as well analyzed in studies of patients with chronic inflammatory diseases (Table 1). In patients with inflammatory bowel disease, Brenner and colleagues reported a strong positive association between systemic GC use and adverse outcomes, with GC treatment as a risk factor for death due to COVID-19 (adjusted OR 11.6, 95% CI 2.09 to 64.74, p = 0.005) [40]. In line with these results, treatment with higher dosages of GC in patients with rheumatic diseases has been shown as an independent risk factor associated with COVID-19-related death [41, 42]. In contrast again, there are studies that do not see any significant correlation in multivariable adjusted analyses [43], or even one report that suggests reduced disease progression from mild to critical conditions under low- to medium-dose GC treatment (5-15 mg/day prednisone) [44]. However, the latter study consisted of only 22 patients with GC therapy.

Overall, there is a growing body of evidence with most studies showing that patients on chronic GC therapy are at higher risk for a SARS-CoV-2 infection and a severe course of disease, regardless of age and comorbidities (Table 1). A significant association between GC treatment and adverse outcomes for COVID-19 was also shown in the meta-analyses mentioned above [28]. In many studies patients with high-dose GC therapy were at particularly high risk for a severe course of disease, so it is reasonable to assume that there is a dose-dependent effect. This data supports the hypothesis, consistent with prior literature, that chronic GC exposure in auto-inflammatory conditions is associated with infectious complications including an adverse outcome after contracting SARS-CoV-2. However, since uncontrolled inflammatory disease activity also enhanced the risk of infection, and disease activity itself was already associated with an increased risk of hospitalization and adverse outcome [41], abrupt discontinuation of GC treatment must be avoided. Especially, as there is a high risk of HPA axis suppression here. Rather, the lowest possible dose is suggested [35].

## Risk in endogenous glucocorticoid excess

Endogenous CS leads to a variety of alterations in the immune system and clinical complications such as sepsis and opportunistic infections. The prevalence of infections is increased to 21-51%, as a result of the excessive exposure to endogenous GCs [45, 46]. The combination of a generalized immunosuppression with impaired immune response and a chronic low-grade inflammatory state is suspected to be responsible for the large number of clinical complications [47]. Pre-existing chronic inflammatory conditions are known to increase the risk of COVID-19-related death [10]. Little is known about the risk of SARS-CoV-2 infection or severe disease progression in patients with endogenous CS. Table 2 summarizes the studies and case reports on patients with endogenous CS. Belaya and colleagues recently reported on three patients with ACTH-dependent CS and confirmed SARS-CoV-2 infection [48]. The course of these three patients ranged from asymptomatic to lethal. As the course was particularly severe in a patient with newly diagnosed and florid CS, the authors assumed that the clinical course of COVID-19 might be dependent on severity of hypercortisolism in patients with CS, and that florid CS and COVID-19 are more likely to require emergency care. Two additional manuscripts report single case studies (Table 2): the first one was a 71-year-old man with florid hypercortisolism and past metyrapone therapy, who recovered after one week of isolation [49], the second a 27-year-old woman who was scheduled for pituitary surgery due to Cushing’s disease when contracting SARS-CoV-2 [50]. The latter developed COVID-19 pneumonia with a respiratory deterioration and the need for 7 L/min oxygen supply by mask, without further risk factors associated with severe COVID-19. After controlling hypercortisolism by a ‘block and replace’ regime with steroidogenesis inhibitors and hydrocortisone, and a further supportive therapy, the patient improved and was successfully operated one month later. The authors concluded from their case that a multi-disciplinary management with prompt treatment strategies was essential, and that endogenous GC excess could have enhanced the severity of SARS-CoV-2 infection [50].

**Table 2:** Patients with endogenous Cushing’s syndrome and SARS-CoV-2 infection

First author	Year of publication	Patient cohort	Number of patients	Number of hospitalized patients	Risk for infection / hospitalization / severe outcome	Key messages
Belaya et al. [48]	2021	Patients with active CS	22 with active CS, of whom 3 had confirmed SARS-CoV-2 infection	3	• One 71-year-old woman with newly diagnosed CS (late-night serum cortisol >1750nmol/L, LNSC 908.6nmol/L) developed dyspnea and died from hemorrhagic pneumonia 7 days later • One 38-year-old woman with a 5-year medical history of active pituitary CS (late-night serum cortisol 581.3nmol/L, 24hUFC 959.7nmol/24h) suffered from dyspnea, cough, fever and chest pain; oxygen therapy, antibiotics and symptomatic treatments lead to full recovery 24 days later • One 66- year-old woman with a 4-year medical history of mild Cushing’s disease (late-night serum cortisol 420.2 nmol/L, LNSC 10.03nmol/L) tested positive for COVID-19 in routine screening and remained asymptomatic	• Severe CS affected by COVID-19 is more likely to require emergency care despite a lack of clinical presentations and low inflammation biomarkers • It seems possible that the clinical course of COVID-19 is dependent on the severity of endogenous hypercortisolism
Serban et al. [49]	2020	Patients with Cushing’s disease	61 with confirmed Cushing’s disease (15 with active disease, 28 in remission with hypoadrenalism, 18 eucortisolemic), of whom 2 (3.2%) had confirmed SARS-CoV-2 infection	1	• One male patient with active CS, obesity, hypertension, dyslipidaemia; discontinued adrenostatic therapy with metyrapone one month before because of side effects; his general state improved after one week of isolation in the domestic environment • One female patient in remission, with hypoadrenalism adequately treated, 55-years, end-stage chronic kidney disease and malnutrition, died after 6 days of hospitalization	• 3.2% of Cushing’s disease patients had a confirmed SARS-CoV-2 infection, compared with 0.6% in the general population in Lombardy at that time • Chronic hypercortisolism may be associated with more serious SARS-CoV-2 infection • Active Cushing’s disease patients should be considered as a fragile population
Yuno et al. [50]	2020	Patient with active Cushing’s disease	1 with active Cushing’s disease and COVID-19	1	• One female patient with active Cushing’s disease and COVID-19 pneumonia, 27-years old, need of 7 L/min oxygen supply by mask, medical treatment with steroidogenesis inhibitors and hydrocortisone (“block and replace”); COVID-19 pneumonia improved with multi-modal treatment; successful trans-sphenoidal surgery was performed one month later after a negative SARS-CoV-2 test result	• Hypercortisolism due to active Cushing’s disease may worsen a SARS-CoV-2 infection • Multi-disciplinary management is mandatory in such cases

*CS* Cushing’s syndrome, *SARS-CoV-2* severe acute respiratory syndrome coronavirus 2, *COVID-19* coronavirus disease-19, *LNSC* late-night salivary cortisol, *24hUFC* 24-hour urinary free cortisol

Table 3 shows published data on serum cortisol concentrations in patients with SARS-CoV-2 infection. Tan and colleagues reported plasma cortisol concentrations in patients hospitalized for COVID-19 within 48 hours after admission [51]. Patients with confirmed COVID-19 had higher plasma cortisol concentrations compared to patients without COVID-19, and cortisol concentrations were associated with a higher mortality in patients with COVID-19 (Table 3) [51, 52]. There is also the hypothesis that endogenous cortisol acts through the activation of unprotected mineralocorticoid receptors and exhibits deleterious effects in COVID-19 disease via this mechanism [53, 54]. As dexamethasone, unlike cortisol, binds more selectively to the glucocorticoid receptor, suppresses the secretion of endogenous cortisol and exhibits only a weak mineralocorticoid effect, it has been suggested that this could be an additional effective action of dexamethasone in critically ill COVID-19 patients, beside its anti-inflammatory action. Simultaneous medication with mineralocorticoid receptor antagonists was suggested to be evaluated in these patients [53, 55]. In line with that, glucocorticoid resistance has been hypothesized to contribute to morbidity and mortality in COVID-19, mechanistically via a reduced ability to inhibit the inflammation triggered by SARS-CoV-2 [56], or via activation of mineralocorticoid receptors, as known in familial glucocorticoid resistance syndromes [57].

**Table 3:** Serum cortisol concentrations in patients with SARS-CoV-2 infection

First author	Year of publication	Patient cohort	Number of patients	Number of hospitalized patients	Risk for infection / hospitalization / severe outcome	Key messages
Tan et al. [51]	2020	Patients with confirmed diagnosis of COVID-19	403 patients with diagnosis of COVID-19, of whom 112 patients died in the study period	403	• Multivariable analysis showed that a doubling of cortisol concentration was associated with a significant 42% increase in the hazard of mortality	• Patients with COVID-19 mount a marked and appropriate acute cortisol stress response • High cortisol concentrations were associated with increased mortality and a reduced median survival
Ramezani et al. [52]	2020	Patients with confirmed diagnosis of COVID-19	30 hospitalized patients with SARS-CoV-2 infection	30	• Higher serum levels of cortisol were found in non-survival patients vs surviving patients (p=0.017)	• Severe COVID-19 infection outcomes are more prominent at a higher level of serum cortisol

*SARS-CoV-2*, severe acute respiratory syndrome coronavirus 2, *COVID-19* coronavirus disease-19, *vs* versus

### Considerations for patients with endogenous Cushing’s syndrome

In response to the SARS-CoV-2 pandemic, several recommendations for the clinical guidance of patients with endogenous CS were published [45, 58–60]. These include preventive factors such as video consultations and adherence to social distancing to reduce the risk of infection, as well as diagnostic and treatment recommendations when extensive differential diagnostic testing and/or surgery is not possible [58]. Surgery is the treatment of choice in all causes of CS, but during the pandemic a delay might occur or be appropriate [59]. Therefore, cortisollowering bridging treatment with steroidogenesis inhibitors are recommended in such situations [45, 58, 59]. Due to the fast onset of action and good effectiveness in controlling hypercortisolism, ketoconazole, metyrapone and osilodrostat should be preferred in florid CS for drug therapy during the pandemic [58, 61]. A medical treatment to control hypercortisolism in patients with CS is recommended not only to prevent disease progression but also to correct hypercoagulability and minimize the risk of COVID-19 [50, 58]. Since higher levels of cortisol in 24-hour urine collection are associated with an increased risk of infection, the magnitude of elevation should be taken into account in the therapy concept [59].

As patients with florid CS are immunocompromised, the febrile response in the case of a SARS-CoV-2 infection might be limited. Furthermore, it is suggested that dyspnea might be enhanced due to left ventricular cardiomyopathy and thoracic muscle weakness in case of COVID-19 pneumonia [45]. In hospitalized patients, prolonged antiviral treatment and empirical prophylaxis with broad-spectrum antibiotics should be considered, because of the increased risk of secondary infections (Table 4). Moreover, both florid CS and COVID-19 are associated with hypercoagulability [62–64]. Thus, patients with CS and COVID-19 might have a higher risk of thromboembolism, which is why treatment with low-molecular-weight heparin is recommended, especially for hospitalized, moderately to severely ill patients [45, 58]. In the case of COVID-19 in a patient with pre-existing adrenostatic therapy, stress doses of GCs should be given to prevent critical illness-related corticosteroid insufficiency [58]. There is also a clinical case of a 67-year-old man with Cushing’s disease receiving pasireotide and metyrapone, who developed adrenal insufficiency during COVID-19 infection requiring GC replacement therapy [65]. ‘Block and replace’ regimens can be useful in certain situations [58, 59].

### Hypercortisolism-associated comorbidities per se might increase the risk for a severe course of disease

Already in early observational studies it was shown that in patients with obesity, cardiovascular diseases, hypertension, immunodeficiency or diabetes COVID-19 can more easily become a critical illness or cause death [66, 67]. All features that are common in patients with endogenous GC excess [68–70]. The increased cardiovascular risk and the increased susceptibility to severe infections are two leading causes of death in patients with CS [71, 72]. Beside the mentioned immune impairments, GC-induced cardiometabolic changes are common and might additionally increase the risk of a severe course of COVID-19. Table 4 shows known hypercortisolism-associated comorbidities and concomitant risk factors for COVID-19.

Regarding GC-induced comorbidities like hypertension, diabetes, cardiovascular diseases and dyslipidemia, close monitoring and optimization of medical treatment are recommended to improve the eventual clinical course of COVID-19 (Table 4) [45]. Even after successful cure of endogenous CS, patients continue to have increased cardiovascular morbidity and mortality [73, 74]. Also, characteristic features and metabolic comorbidities of endogenous CS like cognitive impairments, obesity, diabetes and muscle weakness were reported to partly persist in the long-term despite biochemical remission [72, 75–78]. Since these conditions increase the risk of COVID-19 with a severe course of disease, even patients in remission for CS should be considered as high-risk population, and GC-associated comorbidities should be treated consistently [45].

**Table 4:** Hypercortisolism-associated comorbidities and the risk of COVID-19

Comorbidity in endogenous CS	Prevalence in CS [46, 114, 115]	Reported risk factor in COVID-19	Management recommendations during SARS-CoV-2 pandemic [45, 58, 61]
Arterial hypertension [69]	58-85 %	Zhou et al. [2]	• Hypertensive patients with CS should be considered at high risk for severe COVID-19 • Optimization of medical treatment is recommended to improve the eventual clinical course of COVID-19
Diabetes mellitus [70]	20-47 %	Corona et al. [9]	• Optimization of medical treatment is recommended to improve the eventual clinical course of COVID-19
Hypercoagulability [63]	54 %	Levi et al. [62]	• Strict monitoring of coagulation parameters • Treatment with low-molecular-weight heparin is recommended, especially for hospitalized, moderately to severely ill patients
Immune impairments [45]	21-51%	Wu et al. [116]	• Due to a poor immune response, febrile response in the early phase might be limited • Because of the increased risk of secondary infections, prolonged antiviral treatment and empirical prophylaxis with broad-spectrum antibiotics should be considered in hospitalized cases of COVID-19 and florid CS
Obesity [117]	32-41 %	Rottoli et al. [118]	• Patients with CS and visceral obesity should be carefully monitored in case of COVID-19, due to an increased morbidity and mortality
Atherosclerosis/ cardiovascular diseases [68]	27-31 %	Zou et al. [8]	• Optimization of medical treatment is recommended to improve the eventual clinical course of COVID-19
Myopathy [75]	60-82 %	De Giorgio et al. [119]	• Dyspnea might be enhanced due to left ventricular cardiomyopathy and/or thoracic muscle weakness in patients with CS
Dyslipidemia [120]	38-71 %	Zuin et al. [121]	• Optimization of medical treatment is recommended to improve the eventual clinical course of COVID-19
Mental illness [122]	50-81 %	Barcella et al. [123]	• As mental illness is associated with an unfavorable outcome in patients with COVID-19, neuropsychiatric disorders in patients with CS should be considered and adequately treated
Obstructive sleep apnea [115]	50 %	Strausz et al. [124]	• Consistent therapy is recommended to improve the risk profile and prevent secondary consequences such as hypertension, reduced physical performance and further systemic effects

*CS* Cushing’s syndrome, *SARS-CoV-2* severe acute respiratory syndrome coronavirus 2, *COVID-19* coronavirus disease-19

## SARS-CoV-2 Infection and the Hypothalamic-Pituitary-Adrenal Axis

Little is known about the effects of COVID-19 on the HPA axis. In general, endogenous GCs are essential for survival under conditions of stress [79]. GCs from the adrenal glands have profound metabolic, cardiovascular and immunological roles in an adequate stress response, elevated cortisol concentrations in critically ill patients are triggered by an activation of the HPA axis [80, 81]. Studies on the impact of COVID-19 on endocrine organs are limited. However, because of the expression of angiotensin-converting enzyme 2 (ACE2) in many endocrine glands [82, 83], endocrine organs are likely to be involved in COVID-19 [6, 84]. Alzahrani and colleagues reported on an impaired adrenocortical response in 28 patients with COVID-19 with plasma cortisol and ACTH concentrations indicating central adrenal insufficiency [85]. The authors found an inverse correlation between disease severity and cortisol or ACTH concentrations. In accordance with these data, compared to non-COVID-19 critically ill patients, lower cortisol concentrations were reported in critically ill patients with COVID-19, among whom 67% met the criteria for the diagnosis of critical illness-related corticosteroid insufficiency [83]. Contradicting with these findings is the above-mentioned report of Tan and colleagues on higher cortisol concentrations in patients with COVID-19 [51].

Autopsy studies showed frequent microscopic adrenal lesions in patients with severe COVID-19 [86, 87]. However, whether these led to altered cortisol dynamics or an insufficiency is questionable. Already in the context of the previous outbreak of SARS 2003, it has been hypothesized that SARS-CoV inhibits the adrenal stress responses causing a relative adrenocortical insufficiency, via molecular mimicry of ACTH and an immune response that cross reacts with ACTH, and/or direct hypothalamic and pituitary effects [84, 88]. Data from the previous SARS outbreak even suggested that a substantial proportion of patients (39%) who recovered from the infection had evidence of central adrenal insufficiency 3 months after recovery. As the HPA axis function of the majority recovered within a year, the authors suggested a reversible hypophysitis or direct hypothalamic injury induced by the virus [89]. Whether the beneficial effects of dexamethasone in the treatment of COVID-19 is due to general supportive benefits of GCs in critical ill patients, mitigation of the cytokine release syndrome and ARDS [12, 90], the treatment of undiagnosed adrenal insufficiency [83, 85], or the suppression of endogenous cortisol secretion with reduced effects on mineralocorticoid receptors [53], has to be clarified in the future.

## Glucocorticoids and SARS-CoV-2 mRNA vaccines

Whether patients with endogenous hypercortisolism or GC treatment have a blunted messenger RNA (mRNA) vaccine response is not known. Systemic GC therapy affects both the innate and adaptive immunity. Because of the immunosuppressive actions and the known glucocorticoid receptor expression on all immune cells, the primary concern regarding SARS-CoV-2 vaccines in the setting of GC excess is efficacy. GCs are known to suppress the ability of antigen presenting cells to process antigen and to impair T cell activation [91]. In animal models, GC exposure was also shown to influence B cell function [92]. Furthermore, there is some evidence suggesting that chronic GC treatment may decrease B-cell counts and specific antibody responses in human [93]. Studies on the functional vaccine outcomes in patients with chronic high-dose GC therapy point toward a potentially impaired vaccine-based immunity with decreased serologic responsiveness in individuals with exogenous GCs [94–96]. However, the decrease of vaccine efficacy in these setting was small, and it has been rather established that patients with chronic GC treatment generate an adequate humoral response to vaccines [97–100].

The use of intraarticular corticosteroids was previously associated with an increased risk for developing influenza despite vaccination [101]. Due to the long-lasting systemic effects of intraarticular corticosteroid injections and the time to establish an effective response of the adaptive immune system, it is suggested to conduct an elective corticosteroid injection no less than two weeks prior and one week following mRNA vaccine dose, whenever possible [102, 103]. However, on the other hand, there are also studies that do not see any connection between vaccine responsiveness and short-term systemic GC administration [100, 104, 105]. To the best of our knowledge, there are no available studies on vaccine response in patients with endogenous GC excess. In dogs with hyperadrenocorticism treated with trilostane, the immune response to canine parvovirus vaccination was comparable with that of healthy dogs [106].

Patients with chronic immunosuppressive therapies have been excluded from the large studies of SARS-CoV-2 mRNA vaccines to date. In a recently published prospective study with 436 transplant recipients and immunosuppressive therapy including tacrolimus (83%), corticosteroids (54%), mycophenolate (66%), azathioprine (9%), sirolimus (4%), and everolimus (2%), antibodies were detectable in only 76 of 436 participants (17%, 95% CI 14-21%) at a median of 20 days after the first dose of SARS-CoV-2 mRNA vaccine [107]. This is in contrast with the robust early immune response observed in the SARS-CoV-2 mRNA vaccine trials with 100% seroconversion within 2-3 weeks after immunization with mRNA-1273 and BNT162b2 [108, 109]. Despite limitations, such as antibody measurements after only one dose, this study shows that immunocompromised individuals may show a reduced response to mRNA vaccination [107], and could therefore be at a higher risk. Both the mRNA-1273 and BNT162b2 vaccine trials allowed GC use under an oral prednisone equivalent dose of 20 mg per day for up to 14 days, or 280 mg of prednisone equivalent in total [108, 109]. However, so far, no subgroup analysis was provided with participants who were exposed to GCs. Further studies are required for exogenous GCs and other immunosuppressive agents to determine whether these patients can build up a serum titer of anti-SARS-CoV-2 antibodies comparable with individuals not taking immunosuppressive agents. To answer this question, several studies on efficacy and safety of SARS-CoV-2 mRNA vaccination in specific patient populations with immunomodulatory therapies are currently recruiting or planned (ClinicalTrials.gov: amongst others, NCT04848493, NCT04839315, NCT04845997, NCT04824651, NCT04798625).

Noteworthy, all vaccines approved or currently in late-stage development, including vaccines based on mRNA, are considered inactivated vaccines. With regard to live vaccination, there are safety concern with systemic GC doses equivalent to 2 mg/kg or a dose of 20 mg per day of prednisone equivalents for 2 or more weeks [100, 110, 111]. However, non-live vaccines can be used without restrictions in patients under immunosuppressive therapy [112].

A potentially decreased response to vaccination should not lead to a decision not to vaccinate, and vaccines should not be deferred in these vulnerable patients. As patients with endogenous or exogenous GC excess might be at higher risk for a severe course of COVID-19, immunization is recommended. Thus, it can be assumed that in patients with a higher risk, vaccination even with the result of a lower titer of antibodies is better than no vaccination. Depending on further studies on the immune response, repeated immunization after the period of altered immunocompetence, such as florid endogenous CS or high-dose GC treatment, may be necessary after achieving remission.

## Summary and Conclusion

There is growing evidence that patients under especially high doses of exogenous GC or during endogenous GC excess exhibit an increased risk for SARS-CoV-2 infection and for a severe course of COVID-19. Therefore, in patients with higher doses of exogenous GCs, the lowest possible dose is recommended to control the underlying inflammatory disease. In patients with endogenous CS, adequate control of hypercortisolism is recommended, as increased risk has been associated with the level of cortisol excess. Moreover, good monitoring of metabolic and cardiovascular comorbidities seems particularly important to ameliorate the risk profile of patients with GC excess.

Many questions about the relation between SARS-CoV-2 and endogenous or exogenous GC excess remain unanswered. Further studies are required to understand the risk of these patients as well as the long-term effects of COVID-19 in this population. The immediate collection of data in the SARS-CoV-2 pandemic is important to answer key pressing questions. International collaboration between specialists appears to be of great importance for certain patient groups in order to obtain valid data [113]. However, current limitations should be taken into account in the interpretation of studies and case reports. In particular selection bias and confounding are important to consider [39]. We want to acknowledge that our recommendations are based on the aforementioned and currently available data and evidence, and that it has to be updated based on ongoing data.
